# Steering self-organisation through confinement

**DOI:** 10.1039/d2sm01562e

**Published:** 2023-02-06

**Authors:** Nuno A. M. Araújo, Liesbeth M. C. Janssen, Thomas Barois, Guido Boffetta, Itai Cohen, Alessandro Corbetta, Olivier Dauchot, Marjolein Dijkstra, William M. Durham, Audrey Dussutour, Simon Garnier, Hanneke Gelderblom, Ramin Golestanian, Lucio Isa, Gijsje H. Koenderink, Hartmut Löwen, Ralf Metzler, Marco Polin, C. Patrick Royall, Anđela Šarić, Anupam Sengupta, Cécile Sykes, Vito Trianni, Idan Tuval, Nicolas Vogel, Julia M. Yeomans, Iker Zuriguel, Alvaro Marin, Giorgio Volpe

**Affiliations:** a Departamento de Física, Faculdade de Ciências, Universidade de Lisboa 1749-016 Lisboa Portugal nmaraujo@fc.ul.pt; b Centro de Física Teórica e Computacional, Faculdade de Ciências, Universidade de Lisboa 1749-016 Lisboa Portugal; c Department of Applied Physics and Science Education, Eindhoven University of Technology, P.O. Box 513 5600 MB Eindhoven The Netherlands l.m.c.janssen@tue.nl; d Institute for Complex Molecular Systems, Eindhoven University of Technology, P.O. Box 513 5600 MB Eindhoven The Netherlands; e University of Bordeaux, CNRS, LOMA, UMR 5798 F-33400 Talence France; f Department of Physics and INFN, University of Torino, via Pietro Giuria 1 10125 Torino Italy; g Kavli Institute at Cornell for Nanoscale Science, Cornell University Ithaca New York USA; h Laboratory of Atomic and Solid-State Physics, Cornell University Ithaca New York USA; i Gulliver UMR CNRS 7083, ESPCI Paris, Université PSL 75005 Paris France; j Soft condensed matter, Department of Physics, Debye institute for Nanomaterials Science, Utrecht University, Princetonplein 1 3584 CC Utrecht The Netherlands; k Department of Physics and Astronomy, University of Sheffield Hounsfield Road Sheffield S3 7RH UK; l Research Centre on Animal Cognition (CRCA), Centre for Integrative Biology (CBI), Toulouse University, CNRS, UPS Toulouse 31062 AD France; m Department of Biological Sciences, New Jersey Institute of Technology Newark NJ 07102 USA; n Department of Applied Physics and J. M. Burgers Center for Fluid Dynamics, Eindhoven University of Technology, P.O. Box 513 5600 MB Eindhoven The Netherlands; o Max Planck Institute for Dynamics and Self-Organization (MPI-DS) 37077 Göttingen Germany; p Rudolf Peierls Centre for Theoretical Physics, University of Oxford Oxford OX1 3PU UK; q Laboratory for Soft Materials and Interfaces, Department of Materials, ETH Zürich 8093 Zürich Switzerland; r Department of Bionanoscience, Kavli Institute of Nanoscience, Delft University of Technology 2629 HZ Delft The Netherlands; s Institut für Theoretische Physik II: Weiche Materie, Heinrich-Heine-Universität Düsseldorf, Universitätsstrasse 1 40225 Düsseldorf Germany; t Institute of Physics & Astronomy, University of Potsdam, Karl-Liebknecht-Str 24/25 D-14476 Potsdam-Golm Germany; u Mediterranean Institute for Advanced Studies, IMEDEA UIB-CSIC, C/Miquel Marqués 21 07190 Esporles Spain; v Department of Physics, University of Warwick, Gibbet Hill road CV4 7AL Coventry UK; w Institute of Science and Technology Austria 3400 Klosterneuburg Austria; x Physics of Living Matter, Department of Physics and Materials Science, University of Luxembourg, 162 A, Avenue de la Faïencerie L-1511 Luxembourg; y Laboratoire de Physique de lÉcole normale supérieure, ENS, Université PSL, CNRS, Sorbonne Université, Université Paris Cité F-75005 Paris France; z Institute of Cognitive Sciences and Technologies, CNR, Via San Martino della Battaglia 44 00185 Rome Italy; a Institute of Particle Technology, Friedrich-Alexander Universität Erlangen-Nürnberg, Cauerstrasse 4 91058 Erlangen Germany; b Departamento de Física y Matemática Aplicada, Facultad de Ciencias, Universidad de Navarra Pamplona Spain; c Physics of Fluids Group, Mesa+ Institute, Max Planck Center for Complex Fluid Dynamics and J. M. Burgers Center for Fluid Dynamics, University of Twente 7500AE Enschede The Netherlands a.marin@utwente.nl; d Department of Chemistry, University College London 20 Gordon Street London WC1H 0AJ UK g.volpe@ucl.ac.uk

## Abstract

Self-organisation is the spontaneous emergence of spatio-temporal structures and patterns from the interaction of smaller individual units. Examples are found across many scales in very different systems and scientific disciplines, from physics, materials science and robotics to biology, geophysics and astronomy. Recent research has highlighted how self-organisation can be both mediated and controlled by confinement. Confinement is an action over a system that limits its units’ translational and rotational degrees of freedom, thus also influencing the system's phase space probability density; it can function as either a catalyst or inhibitor of self-organisation. Confinement can then become a means to actively steer the emergence or suppression of collective phenomena in space and time. Here, to provide a common framework and perspective for future research, we examine the role of confinement in the self-organisation of soft-matter systems and identify overarching scientific challenges that need to be addressed to harness its full scientific and technological potential in soft matter and related fields. By drawing analogies with other disciplines, this framework will accelerate a common deeper understanding of self-organisation and trigger the development of innovative strategies to steer it using confinement, with impact on, *e.g.*, the design of smarter materials, tissue engineering for biomedicine and in guiding active matter.

## Abstract 2

In this perspective article, we discuss how soft matter can spearhead efforts to harness confinement as a tool to steer self-organisation across different scales and disciplines.

## Graphical abstract

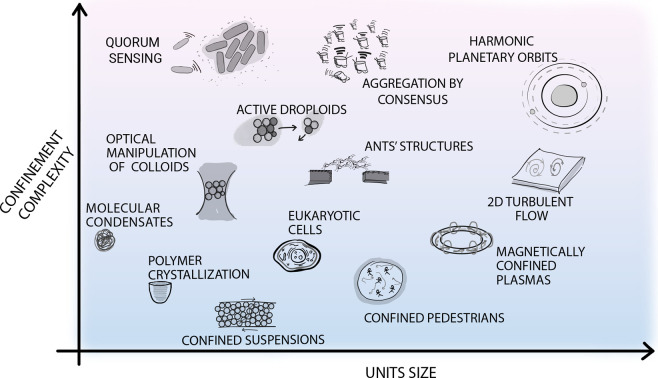

## 1 Introduction

From molecular aggregates^1^ to groups of animals^2^ and human crowds,^3,4^ from microswimmers^5^ to granular materials^6^ and robotic swarms,^7^ examples of systems that self-organise can be found across a wide diversity of length and time scales.^8,9^ The concept of self-organisation in soft matter and related fields came to the fore in the 20th century^10^ and defines the spontaneous emergence of large-scale collective structures and patterns in space and/or time from the interaction of many individual units,^8,9^ such as molecules, colloidal particles, cells, animals, robots, pedestrians or even astronomical objects. These units can be highly heterogeneous in size, shape, composition and function (as is often the case in biological systems) or largely identical (as in monodisperse colloidal dispersions). The units can also be active (*e.g.* molecular motors, cells, animals and pedestrians) or passive (*e.g.* colloids, granular matter and planets), depending on whether they can or cannot transform available energy to perform work at the level of the individual units.

There are two key features of self-organisation that deserve to be highlighted: first, the self-organised structures extend over much larger length scales than the size of the individual units; second, these structures yield emergent properties and functions, beyond what is achievable by their constituent units alone.^11^ This emergence of non-trivial, non-additive collective features on large scales is what makes the topic of self-organisation fascinating. On the one hand, it captures how complex behaviour can develop and evolve from simple units, *e.g.* life itself emerged from a cocktail of lifeless molecules.^12^ On the other hand, it provides inspiration to materials scientists and system engineers, who aim to mimic this spontaneous complexity to revolutionise man-made materials and devices.^13^

**Fig. 1:**
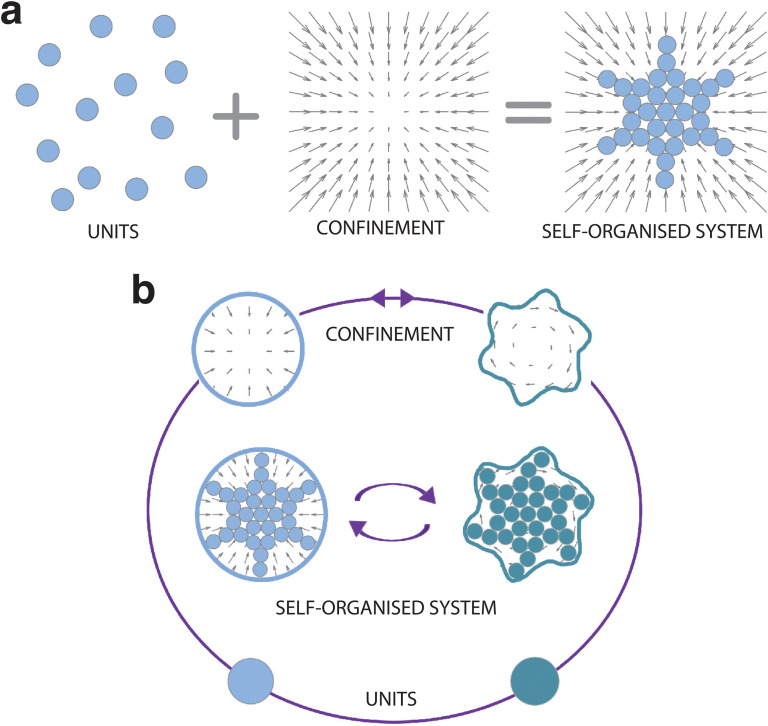
Emergence of structure from confined self-organising units. (a) Self-organisation is the emergence of large-scale structures and patterns from individual units. Confinement can act as a catalyst (as in the diagram) or inhibitor for a self-organising system. The arrows represent an external force field acting on the units. (b) Steering self-organisation through confinement requires encoding feedback loops in the process so that units and/or confining elements can adapt and evolve with the self-organising system. In the schematics, this is visualised by a change in both the confinement (solid lines and corresponding force field) and the units (here symbolically represented by a change in colour). As a consequence the emerging self-organisation patterns differ.

**Fig. 2:**
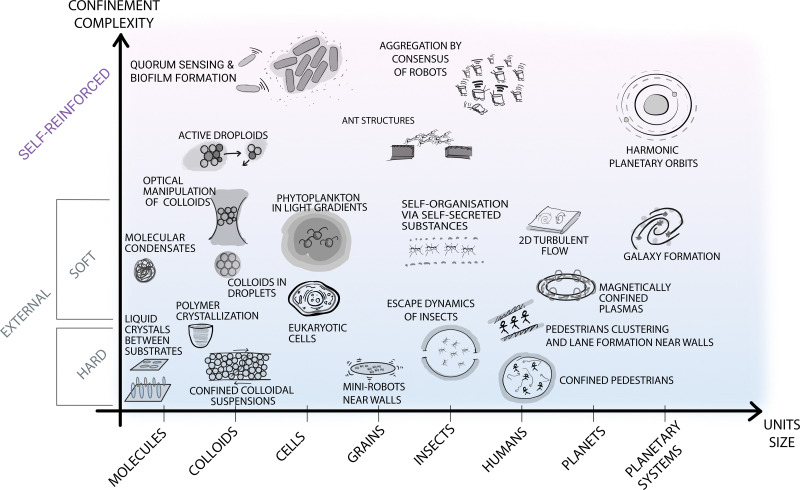
Self-organisation at various length scales under different types of confinement. The diagram contains selected examples of self-organisation under different types of confinement occurring at different spatial (and time) scales in both natural and man-made systems. The horizontal axis represents the length scale of the self-organising units, from molecular up to astronomical scales. The vertical axis represents the type of confinement ordered based on its complexity and ability to be readily parameterised. At the bottom of the diagram, simpler and better understood forms of confinement are highlighted with blue shading. These include external boundaries and fields, *e.g.*, small robots near walls or the gravitational field confining Earth's atmosphere for turbulent flows.^17^ At the top of the diagram, a different type of confinement is purposely separated from the other examples, as less understood but potentially more promising to steer self-organisation. These forms of confinement include feedback loops between the self-organising units and the confining features (*e.g.* in the quorum sensing that induces biofilm formation in microbes,^24^ in the information exchange among ants to generate structures such as bridges,^25^ or in the self-induced gravitational attraction that leads to harmonic orbit resonances^26^).

It is now widely recognised that confinement can influence and even steer the self-organisation process (Fig. 1). Here we take a rather broad definition of confinement, *i.e.* a constraint in the translational and rotational degrees of freedom of the units that alters the phase space probability density. In soft matter, such confinement usually stems, *e.g.*, from the presence of surfaces, interfaces, fields, potentials and flow. In other disciplines, confinement can also be induced by less tangible constraints, such as psychological barriers identified in animal and crowd dynamics.^14^ The variety of self-organising systems influenced by confinement is indeed immense, spanning a very wide range of length scales (Fig. 2): from active filaments driven by microscopic molecular motors^15^ or molecular condensates^16^ enclosed within living cells, to the emergence of macroscopic coherent flow structures confined by Earth's atmosphere,^17^ to the formation of entire galaxies under the pull of the gravitational potentials of black holes.^18^ While confinement is not always required for a system to self-organise,^19^ it can play a pivotal role as either a catalyst or inhibitor for self-organisation. In this regard, one of the most promising applications of confinement in self-organisation is to employ it as a control knob at the hand of researchers and engineers to tune the emergence of collective phenomena. For example, applications of this principle can already be found in the design of scaffolds for tissue engineering,^20^ of the features of a polymer melt for nanolithography and coating methods,^21,22^ and of crowd management strategies *via* the use of physical barriers.^23^

Here, we argue that confinement can be designed to actively steer self-organisation. To achieve this goal, a concerted effort across disciplines is needed. So far, efforts to understand and control self-organisation under confinement have been siloed, focusing mainly on specific systems in isolation, such as colloids,^5^ cells^27^ and pedestrians.^23^ However, there are many analogous questions and technical challenges found across multiple scales, systems and disciplines, which need to be addressed systematically before the full potential of confinement is harnessed to actively steer self-organisation. In some fields the understanding of the topic stems mainly from theoretical results (*e.g.* in particle and plasma physics), due to the very high levels of investment, technical development and human resources required to access the relevant length, time and energy scales experimentally. By contrast, the characteristic scales of soft matter systems can be readily controlled in experiments, so that soft matter can be employed as a tool for studying self-organisation and controlling it *via* confinement in diverse systems by enabling a unique synergy between theoretical, numerical and experimental groups. We thus propose a common roadmap towards this shared aim based on soft matter. To help translate these ideas to other disciplines, we define a unifying language to discuss confinement in self-organisation. We then identify the most relevant scientific challenges and list the conceptual and technological advances required to tackle them.

## 2 The role of confinement in self-organisation

In some systems, certain types of confinement are a prerequisite for self-organisation to emerge. For example, most of the organelles of a living cell^27^ only develop in the presence of a confining cell membrane, which compartmentalises its interior and separates it from the external world;^28^ intracellular liquid–liquid phase separations drive the formation of molecular condensates in cells, which are crucial for the occurrence of many biological processes, including RNA metabolism, ribosome biogenesis, DNA damage response and signal transduction.^29^ For other systems, different forms of confinement lead to the formation of alternative structures and patterns. For example, surfactant molecules form vesicles in solution^30^ but can also form monolayers on surfaces.^30^

In general, besides directly influencing the translational and rotational degrees of freedom of the units, confinement can affect and steer self-organisation in a number of non-exclusive ways: it can alter the nature and strength of the interactions among them and/or introduce new interactions; it can limit the number and type of units that can interact with each other; it can change the phase space of the self-organising system and its underlying energy landscape; it can induce a symmetry breaking in the system; it can modify the encounter rates between units and the probability for sequential or parallel reactions to take place; finally, it can also enable cross-talks across different scales.

Due to the inherent breadth of the concept of confinement, it is important – but *a priori* difficult – to identify a common language that can facilitate the dialog between soft matter and other disciplines. As a first step towards this goal, we propose here a classification scheme of different types of confinement, which can help to better delineate the key characteristics of the underlying physics and to stimulate cross-fertilisation across fields. Recognising that the multifaceted nature of confinement cannot be captured in a simple binary classification, we here propose a list of non-mutually exclusive classifications of confinement depending on its origin, nature, and effect:

• Hard *vs.* soft. Hard confinement is not affected by the dynamics of the self-organising system (as in the case of a solid wall for soft and active matter^4,31–33^), while soft confinement can deform, reshape, adapt and evolve in response to the dynamics of the self-organisation process (as in the case of flexible membranes^34,35^ or fluid interfaces^36^). Hence, in the latter case, there is a feedback mechanism between the units and the confining boundary, as exemplified by, *e.g.*, stem cells that can change their fate depending on the softness of their confining environment.^37^ In general, soft confinement does not necessarily imply a boundary (*e.g.* a membrane). For example, for active matter systems, it can also stem from an intrinsic capability of the units to sense or perceive their surrounding and respond to it, as in the case of chemical secretions for bacteria^38^ or ants,^39^ in a time-dependent distribution of resources consumed by microswimmers,^32^ and in the communication range for animals and robots.^40^

• Static *vs.* dynamic. Static confinement is invariant in time (*e.g.* the walls of a microfluidic chamber for microswimmers^41^ or the plates used to confine active granular matter^31^); dynamic confinement instead varies in time (*e.g.* time-varying chemical gradients acting as confining fields for groups of cells in tissue,^42^ the remodelling of the extracellular matrix by migrating cells^43^ or cues leading to history-dependent formations for social animals, as in the case of ants following paths previously made by their peers^39^).

• Positively *vs.* negatively reinforcing. Positive and negative reinforcements designate situations where the self-organisation process is enhanced (*e.g.* by autoinducers in microbial quorum sensing^38^ or by chemical gradients in tissue formation and proliferation^44^) or disrupted by the presence of confinement (*e.g.* in the reduction of order in crystal formation due to a porous medium^45^).

• External *vs.* self-reinforced. Finally, confinement is often identified as an external feature, *i.e.* not belonging to the self-organising system. However, taking inspiration from certain fields (*e.g.* in the study of active colloids, social animals, and in swarm robotics), there are also forms of confinement that originate from the system itself – a phenomenon we refer to as self-imposed or self-reinforced confinement. This applies to situations where the constraint originates from within the collective dynamics through internal feedback (*e.g.* perceptual cues for lane formation in social animals, such as ants^39^), as illustrated in the top part of Fig. 2. Such feedback facilitates a completely different type of confinement, the concept of which may also be generalised to other disciplines.

Gaining control over self-organisation through confinement in soft matter and beyond requires the scientific community to leverage the more complex forms of confinement mentioned above, taking advantage of soft, dynamic, and self-reinforced boundaries to create externally or internally imposed feedback mechanisms to steer the emergence or suppression of collective behaviours in a self-organising system (Fig. 2).

## 3 Overarching scientific challenges

Developing the tools to steer self-organisation through confinement requires us first to gain a deeper fundamental understanding across the systems, scales, and disciplines of how confinement promotes or suppresses the emergence of collective patterns in space and time. We have identified five synergistic areas where further knowledge is required to drive the field forward: universality, heterogeneity, hierarchy, reciprocity, and design by confinement. Whilst universality and heterogeneity are challenges shared with self-organisation in general and have been discussed broadly in this context, the focus here is on the role of confinement.

**Fig. 3:**
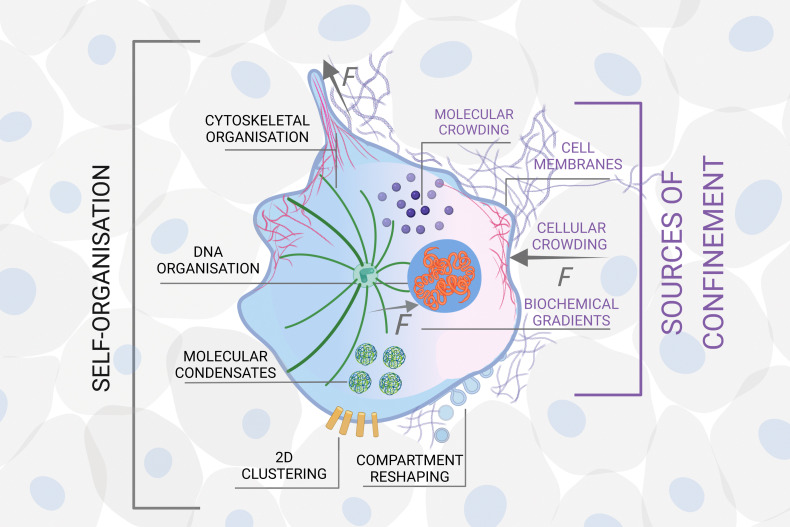
Example of hierarchical self-organisation under confinement in biology. Hierarchical organisation from molecules to tissue *via* the formation of macromolecules, cellular organelles and cells. At each stage, self-organised structures become units for further self-organisation subject to a different type of confinement, here illustrated at the molecular, cellular and tissue scale. Sources of confinement include, *e.g.*, physical boundaries, mechanical forces (*F*) and chemical gradients. The emergence of complex functionality in biological systems relies on the existence of such hierarchical structures. Created with BioRender.com.

• Universality aims at establishing to what extent the patterns observed in a system can be generalised to other systems, scales, and disciplines. Moving forward, it is crucial to identify observable quantities that can help establish if a self-organisation phenomenon is indeed universal or system specific and whether confinement alters this conclusion. Intrinsically, confinement introduces characteristic (length and/or time) scales to the process, thus potentially jeopardising universality across scales. For example, the evacuation of units through bottlenecks mostly follows a common statistical framework regardless of their nature. However, while the presence of an obstacle can help frustrate the formation of arches in granular silos, its efficiency for living systems has not been proven yet, with contradicting results depending on the conditions of the experiments and the properties of the units.^46^ Nonetheless, establishing the conditions under which system-specific observations can be generalised to other systems and disciplines, and establishing robust measures of universality, is pivotal to develop controllable models to steer self-organisation *via* confinement. Experimentally, checking universality across scales requires a *trans*-disciplinary approach, performing experiments with different systems and quantifying them using the same set of measurements. For example, following this approach, it has been recently shown that auto-catalytic growth of aggregates in confining flows displays identical scaling behaviour across more than four orders of magnitude in length, and the interface fluctuations of the growing aggregates obey universal laws.^47^ Theoretical and numerical modelling can also be of help to identify how characteristic scales, specific dynamic rules, and interactions affect universality.

• Heterogeneity addresses how variability in the units (*e.g.* in morphogenesis,^48^ cell differentiation and cancer cells^49^ or in polydisperse colloids^50^) or in the confining element (*e.g.* heterogeneity in both flow and the distribution of chemicals induced by a porous material^51^) influences the emergence of collective behaviour. Specific questions that need to be addressed include how sensitive self-organisation patterns are to variations in size, shape and interactions among the units as well as how confinement can be used to control the level of a system's heterogeneity in space and time taking into account potential system-specific delays. Indeed, most studies of self-organisation in soft-matter systems (*e.g.* colloidal suspensions) have mainly focused on units that are monodisperse in size, shape, and chemical composition, or mixtures of a few species. However, with currently available techniques it is possible to explore how heterogeneity in size, shape, and interaction potential of the units affects the self-organisation process.^52,53^ Furthermore, the heterogeneity of the confinement in space and time can be employed to influence the self-organising units (*e.g.* by promoting their segregation or mixing^54^) and, *vice versa*, confinement can be used to trigger the emergence of heterogeneity in the self-organising system (*e.g.* promoting cell differentiation^49^ or the self-templated assembly of colloidal particles in complex crystal tessellations^55^). For example, due to their small size, colloidal particles can be manipulated with external optical potentials that can vary in space and time.^56,57^ Heterogeneity can be introduced, *e.g.*, by generating disordered optical potentials exploiting the formation of speckle patterns when light propagates through complex media.^58,59^ With the advance of wave modulation techniques, similar experiments could be extended to shorter length scales (*e.g.* electron microscopy)^60^ and larger length scales (*e.g.* acoustics).^61^ Finally, in living systems, the transduction of external stimuli into biological signals that control the behaviour of the units (*e.g.*, cells) goes through biochemical processes that are not instantaneous. Thus, the design of spatial heterogeneity in the confinement elements to control self-organisation needs to account for the timescale over which this adaptation occurs.^62^

• Hierarchy: self-organisation can develop hierarchically, when the confinement at a certain scale defines the units at a larger length scale (Fig. 3). For example, in biological systems, molecules (units) self-organise inside a cell confined by its membrane.^63^ The cells themselves can become the units when they form tissues and organs, confined, *e.g.*, by the extracellular matrix.^64^ Tissues and organs define living entities which can go on to form flocks, herds, schools, confined, *e.g.*, by feedback from their senses and perception.^65^ These groups of animals can then form entire ecosystems confined by their local geography distribution.^66^ In these hierarchical structures, the confining elements at different scales mediate bidirectional (usually non-reciprocal) interactions and flow of information from smaller to larger scales, and *vice versa*. For instance, in biology, the cell membrane is the key confining entity for intracellular self-organisation, but at the same time it defines the cell as an individual unit for multicellular organisation of tissues and organs, thus enabling complex functionalities to emerge. Importantly, the shape and chemical composition of the cell membrane is continuously evolving due to both mechanical and chemical stimuli from the surrounding tissue^67^ and from the cell's interior, thus acting as a mediator of the feedback between different scales. The overarching key challenge here is to elucidate, measure and model how (and when) confinement at different scales mediates or separates the cross-talk and interdependence between scales. Studies conducted at the interface between soft matter, active matter and biology are ideally suited to shed light on this challenge due to the intrinsic hierarchy, *e.g.*, in biological tissue. For example, swimming starfish embryos self-organise into active chiral crystals with odd elastic responses that persist for several hours.^68^ To design synthetic materials that show a similar unique blend of functionality and structure, one needs a fundamental understanding of how these large structures emerge and how they redefine the activity of the individual units. Hierarchy can then become a design strategy for a material's self-assembly: for example, interfacial confinement has been used both to spontaneously assemble supracolloidal building blocks and to further organise them into hierarchically structured materials, thus adding layers of self-organisation within the same colloidal system.^69^

• Reciprocity can be defined as the formation of dynamic feedback loops between units and soft confinement, leading to adaptation, responsiveness and even evolution of a self-organising system in response to changing environmental conditions. An example is provided by cell-matrix interactions in wound healing and tissue regeneration,^70^ where the extracellular matrix confines cells, forcing them to adopt certain morphologies. Mechanotransduction can then induce cells to secrete collagen aligned with the surrounding extracellular matrix, which then further promotes cell organisation. Understanding the interplay between self-organising units and confinement can address both fundamental questions (*e.g.* is life a product of confinement or *vice versa*?) and help define design rules to steer self-organisation through confinement for applications, such as the development of shape-changing scaffolds to drive the growth of artificial tissues and organs. To study reciprocity, controlled experiments should either change the softness of the confinement, by considering different strengths of response to the behavior of the units (*e.g.* as done for cells on granular beds^71^), or tune the response of the units to external stimuli by, for example, knocking out genes believed to be responsible for a given behaviour, *e.g.*, in bacterial systems.^72^

• Design by confinement: the ultimate challenge is to identify and implement tangible design rules (1) to realise confining features that can lead to the emergence of desired patterns from units with known properties (forward design) or (2) to optimise the units to obtain targeted spatio-temporal structures (inverse design) under different realisations of confinement. For example, in the case of units (*e.g.* active colloids or pedestrians) moving near confining features, the realisation of asymmetrically shaped walls can be used to organise their flow in opposite directions through a corridor (forward design),^73^ while crowd behaviour can be engineered through the use of smart management tools (*e.g.* dynamic light patterns) in order to redirect pedestrians towards less dense areas (inverse design).^74^ To take full advantage of design by confinement, some open questions need addressing first: What are the key relevant interactions between units and confinement that need to be controlled? How easily can these control knobs be translated into inputs of design rules for self-organisation? Crucially, what minimal information should be encoded in low-level elements (either units or confinement) to direct self-organisation? And how should this be achieved practically in soft matter systems? To enable optimal control, the relevant information indeed needs to be encoded dynamically in both units and confining elements to generate complex, adaptable feedback mechanisms. Biological systems are particularly good at encoding information (*e.g. via* DNA and RNA) in small volumes and dynamically exploiting confinement to create function (*e.g.* by packing DNA in chromosomes within the cell nucleus or by assembling and disassembling functional compartments in cells, such as lysosomes or membrane-less organelles). Microscopic synthetic materials are still far behind their biological counterparts, so that there is broad scope for further developing synthetic materials to mimic the rich information-encoding capabilities of biological structures and harness emergence for technological applications, *e.g.* to develop programmable materials and smart devices for biomedicine^13^ or for crowd management.^23^

## 4 Overarching technical challenges

The above discussion highlights several avenues for future research, which, to be addressed, will require multiple conceptual and technological advancements. While methods and techniques are often system-specific, we expect the following open technical challenges to become relevant across scales and disciplines in the context of steering self-organisation through confinement.

First, we must develop tools to precisely characterise confinement, the interactions among the units, and the emergent structures. Experimentally, the nature and strength of confinement is not always easy to identify or quantify. This becomes particularly challenging for soft confinement (*e.g.* for chemical gradients), moving boundaries due to their time dependence, and self-imposed forms of confinement that are intrinsically difficult to define and probe. Furthermore, the act of measurement might even alter the properties of the confining element itself, as already anticipated by Niels Bohr's complementarity principle for biology.^75^ Similarly, measuring the interactions among the units can pose a major challenge: in tissues for example, cell–cell interactions are influenced by a complex interplay of biochemical and mechanical signalling pathways and even by the constraints imposed by the surrounding medium;^70^ in human crowds and animal groups, the interactions are influenced by psychological and cognitive factors that are difficult to quantify, especially given the intrinsic heterogeneity among individuals;^2,76^ for active systems, making *a priori* assumptions about interactions may not be sufficient and new ways must be devised to characterise interactions from observations, *e.g.* using machine learning approaches.^77^ It can also be extremely challenging to dynamically probe the emerging self-organising structures from the outside: for example, due to partial or total opacity of the boundaries, real-time imaging with light microscopy can be problematic *in vivo*, and the confinement itself can become a barrier to extract information;^78^ in colloidal systems, interactions, while well-understood and measurable in bulk, are strongly affected by and less characterised at interfaces, *e.g.* liquid interfaces;^36^*in vivo* measurements can also be particularly difficult as the techniques used to probe the system can quickly become invasive enough to alter it (*e.g.* the phototoxicity and bleaching caused by fluorescence microscopy^79^).

Second, to develop a deeper understanding of how self-organisation can be steered through confinement, we must learn to identify and harness the key physical features both at a given scale and across scales. Notably, in the context of hierarchical confinement and reciprocity, one must first identify the relevant quantities that dictate the flow of information (*e.g.* pH, concentrations, mechanical forces, fluid velocity, chemical gradients, elasticity, *etc.*) and be able to measure these, before being able to understand the full cross-talk across scales. This is also particularly crucial when we seek to identify ‘universality classes’ of self-organisation under confinement, and to translate the novel concept of self-reinforced confinement to other fields. To this end improved multi-scale and coarse-grained models will be required, the development of which should occur in close synergy with experimental work to validate them. More generally, we must work towards improved experiments and models that are sufficiently simple and well-controlled to allow for scientific interpretation but which are also sufficiently detailed to capture the relevant phenomena observed under real-life conditions. This is imperative if we want to use these models to predict how different types of confinement and tailored units can steer self-organisation.

Lastly, to design by confinement, we must equip both the units and the boundaries with information-encoding and -processing degrees of freedom to enable adaptive feedback mechanisms. Biological systems have mastered the processes required to translate molecular sequences into the functions of life. Recent advances in gene-editing techniques have paved the way to an unprecedented level of external control over cellular pathways, processes and functions.^80^ For example, in bacterial systems, gene editing techniques have allowed researchers to isolate the influence of the confining topology on the emergence of social interactions between cells.^72^ When considering man-made materials, depending on the scales and nature of the system of interest, the fabrication of information-encoding units and confinement is still a technical challenge, which could be overcome with further development of techniques such as genetic engineering for biohybrid machines^81^ as well as a combination of nano-, microfabrication, 3D printing and time-varying external fields for man-made materials, such as colloidal particles^82^ and elastomers.^83^ In fact, in these man-made systems, a key technical challenge is the need for strong miniaturisation (as required by specific applications like precision medicine), which will limit the way we can design and control self-organising units and confinement at the smaller scales in future years. Yet, advances can still be obtained thanks to the rapid progress in the field of machine learning, which is expected to guide the *in silico* exploration of the enormous space of possibilities, both for new materials design (units and boundaries) and for the discovery of new self-organising structures in space and time.^84^ While the required level of miniaturisation might still be out of reach in many experimental man-made microscopic systems, design ideas can be first tested experimentally using soft macro, micro, and nano-robotic systems, due to the ease of programming complex interaction rules,^85^ in order to test if complex strategies designed *in silico* are borne out in an experimental system.

## 5 Conclusion

In conclusion, steering self-organisation through confinement is a very active and rapidly evolving field of research, which is intrinsically multidisciplinary. To push the field forward, the scientific community working on self-organisation should increasingly take advantage of the cross-fertilisation of ideas that results from sharing hypotheses, theoretical approaches and experimental methods among experts from different fields and disciplines (*e.g.* between physical sciences and life sciences, between synthetic and natural systems, between small and large length scales). The field of soft matter, being intrinsically interdisciplinary, has evolved to show an ever-growing synergy among experts from different backgrounds, as observed recently in the field of active matter. A similar synergy can be beneficial to advance the understanding of self-organisation under confinement as a whole across the scales and the disciplines. This cross-communication is *a priori* not easy, as it requires a common language and consensus on key open research questions and objectives. Certainly, the road ahead is still difficult and many steps need to be taken collectively to bring together the broader community, define confinement and its impact on self-organisation incontrovertibly, and, thus, advance the field in a synergistic way. This perspective article provides a first step in this direction mainly based on work from the soft-matter community. We hope that it will serve as an impetus for the broader scientific community to join this collective effort and meet the exciting challenges that are faced across domains, length and time scales by the possibility of steering self-organisation through confinement.

## Author contributions

Conceptualisation: AM, GV. Data Curation: NA, LJ, AM, GV. Formal analysis: NA, LJ, AM, GV. Funding acquisition: NA, LJ, AM, GV. Investigation: all. Methodology: NA, LJ, AM, GV. Project administration: NA, LJ, AM, GV. Supervision: NA, LJ, AM, GV. Validation: NA, LJ, AM, GV. Visualisation: AM, AŠ, GV. Writing – original draft: all. Writing – review and editing: all.

## Conflicts of interest

There are no conflicts to declare.

## Supplementary Material
